# Applying Human-Centered Design Principles to Digital Syndromic Surveillance at a Mass Gathering in India: Viewpoint

**DOI:** 10.2196/27952

**Published:** 2022-01-10

**Authors:** Ahmed Shaikh, Abhishek Bhatia, Ghanshyam Yadav, Shashwat Hora, Chung Won, Mark Shankar, Aaron Heerboth, Prakash Vemulapalli, Paresh Navalkar, Kunal Oswal, Clay Heaton, Sujata Saunik, Tarun Khanna, Satchit Balsari

**Affiliations:** 1 Institute for Critical Care Medicine Mount Sinai Hospital New York, NY United States; 2 Carolina Health Informatics Program University of North Carolina at Chapel Hill Chapel Hill, NC United States; 3 India Digital Health Network Lakshmi Mittal and Family South Asia Institute Harvard University Cambridge, MA United States; 4 Department of Obstetrics and Gynecology Baylor College of Medicine Houston, TX United States; 5 Articulate Labs, Inc San Francisco, CA United States; 6 Department of Emergency Medicine Memorial Hermann Hospital -Baylor College of Medicine Houston, TX United States; 7 Department of Emergency Medicine Jacobi Medical Center New York, NY United States; 8 University Hospitals Center for Emergency Medicine Cleveland Medical Center Cleveland, OH United States; 9 Lifesupporters Institute of Health Sciences Mumbai India; 10 Department of Public Health Dentistry Sharad Pawar Dental College Maharashtra India; 11 Department of General Administration Government of Maharashtra Mumbai India; 12 Harvard TH Chan School of Public Health Boston, MA United States; 13 Harvard Business School Boston, MA United States; 14 Department of Global Health and Population Harvard TH Chan School of Public Health Boston, MA United States; 15 Department of Emergency Medicine Beth Israel Deaconess- Harvard Medical School Boston, MA United States

**Keywords:** mHealth, design, human centered design, intervention, syndromic surveillance, digital health

## Abstract

In the wake of the COVID-19 pandemic, digital health tools have been deployed by governments around the world to advance clinical and population health objectives. Few interventions have been successful or have achieved sustainability or scale. In India, government agencies are proposing sweeping changes to India’s digital health architecture. Underpinning these initiatives is the assumption that mobile health solutions will find near universal acceptance and uptake, though the observed reticence of clinicians to use electronic health records suggests otherwise. In this practice article, we describe our experience with implementing a digital surveillance tool at a large mass gathering, attended by nearly 30 million people. Deployed with limited resources and in a dynamic chaotic setting, the adherence to human-centered design principles resulted in near universal adoption and high end-user satisfaction. Through this use case, we share generalizable lessons in the importance of contextual relevance, stakeholder participation, customizability, and rapid iteration, while designing digital health tools for individuals or populations.

## Introduction

In 2020, the government of India announced the National Digital Health Mission (NDHM), a vision for a federated health information ecosystem that is expected to catalyze India’s digital health transformation [1]. The NDHM distinguishes itself from its predecessors by embracing an enabling platform approach that is expected to allow market solutions like mobile apps and wearables to seamlessly interface with patient health records, opening the possibility of exponential growth in digital solutions in both the public and private sectors [2-4].

Though the proposed open ecosystem provides an unprecedented opportunity for growth and competition in the development of digital health tools, the lack of systematic approaches to evaluate and validate digital health interventions risks diluting the impact of the very large investments expected in this space [5-7]. The vast majority of digital health interventions fail to scale for a variety of reasons, including the lack of human-centered design and poor contextual knowledge [8,9]. These challenges are further exacerbated in low resource settings that suffer from significant institutional voids [10,11]. The near-universal spectacle of overburdened providers entering data in hard-to-navigate survey forms points to the emphasis placed on programmatic and reporting needs, instead of the needs of the key users, namely, patients and health care providers [12,13].

Solutions that have succeeded have demonstrated a commitment to human-centered design and a deep understanding of the infrastructural, social, and economic constraints faced by providers in primary care settings. Programs like Gujarat’s TECHO platform for maternal and child health services in tribal communities and Sangath’s ESSENCE program for training community health workers in mental health have relied on a resource-intensive strategy that combines task-shifting, training, and technology for successful digital health implementation in India [14,15]. These programs adopted iterative ideation, prototyping, and testing cycles early in their product design process [16,17].

In this paper, we describe the successful implementation of a mobile health tool in a dynamic transient setting, the 2015 Nashik Kumbh Mela in India, where the administrators had minimal to no time allocated to training providers, and yet, adoption was near universal. The tool was used by over 100 clinicians to enter data and track performance, and by state administrators to monitor disease outbreaks in real-time [18]. The tool also provided essential data on utilization, resource-allocation, and prescription patterns. We attribute the tool’s success to adherence to human-centered design principles, great emphasis on user experience, meticulous attention to workflow, and strict adherence to data minimization, principles which may serve well the many large-scale digital health rollouts being attempted all over the developing world [19-22]. Through this use case, we share generalizable lessons in the importance of contextual relevance, stakeholder participation, customizability, and rapid iteration, while designing digital health tools for individuals or populations.

## The Nashik Kumbh Mela

The Kumbh Mela is a religious mass gathering that occurs every 3 to 4 years at 1 of 4 pilgrimage sites in India [23]. In 2015, the Kumbh Mela was held in the adjacent towns of Nashik (population: 1,486,053) and Trimbakeshwar (population: 12,056) from August 26 to September 25, 2015, and attended by an estimated 30 million people [24,25]. A span of three 3-day festivities at each site marked the most auspicious days when the majority of pilgrims seek a dip in the holy waters of the Ram Kund (pond) in Nashik or the Godavari river in Trimbakeshwar.

As with all Kumbh Melas, the state government serves as patron and host, providing a range of services to ensure the well-being and safety of the visitors and the local population. Preparations begin months in advance and entail additional transport facilities; staging of incoming traffic away from crowded zones; provision of clean water supply, electricity, and temporary shelter; and construction of over 50 temporary health care facilities along the arterial routes leading to the pilgrimage sites [26,27]. These clinics are staffed by a physician, nurse, and pharmacist, and offer little to no laboratory tests. The concern for stampedes and disease outbreaks is high at the Kumbh Melas, and governments have historically invested significant resources toward mitigating the risks for both [28-32]. Strict water quality monitoring, provision of clean safe drinking water, and provision of health outposts (clinics) have been the norm at these Melas [30]. Physicians posted at these clinics routinely have no more than a couple of minutes to see each patient, and hastily scribble down the patient’s chief complaint on a paper-based log.

In 2015, the Public Health Department, Government of Maharashtra, invited our team of researchers to build on prior experience at the Allahabad Mela, to create a digital disease surveillance system for the 2015 Kumbh Mela [33].

## Designing for Digital Health

We adopted design principles recommended by IDEO’s Field Guide to Human-Centered Design [34]. These principles also appear in the more recently published “Human-Centered Design 4 Health” guidelines by UNICEF and in the World Health Organization’s “Digital Health Implementation Investment Guide” [21,22,35], all of which underscore the importance of an approach and recognize that projects loop through the phases of ideation, inspiration, and implementation multiple times as they incorporate user feedback in every stage until a refined solution is ready for scale [17].

### Step 1: Inspiration

#### Assembling an Interdisciplinary Team

The process of inspiration entails an exploration of the circumstances that motivate the need for a solution [17]. At the start of the design process, we defined the following 2 groups of end users: (1) the clinicians staffing the clinics, who would also be responsible for data entry, and (2) the public health officials charged with managing potential disease outbreaks. We assembled an interdisciplinary team that included senior state officials (to facilitate subsequent approvals) and subject-matter experts, including field researchers, data scientists, clinicians, and medical students with prior experience at the 2013 Kumbh. This team composition ensured the technical team had access to rich contextual intelligence from the early stages of the design process and to senior policymakers.

#### Needs Assessment

**Figure 1:**
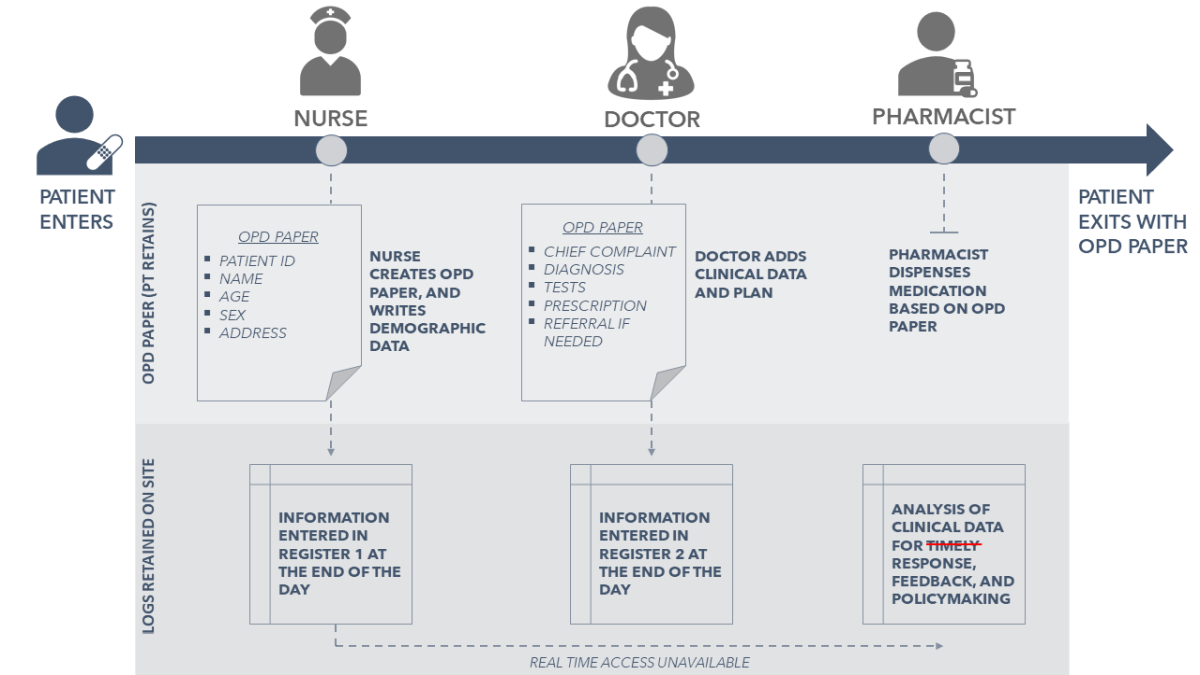
Workflow at the 2015 Kumbh Mela clinics prior to the intervention. OPD: outpatient department.

The research team then carried out a comprehensive review of prior research and related literature; conducted a series of key informant interviews with local and state public health leadership and medical officers; and performed site visits to understand the layout of the clinics, workflow, potential pitfalls, and day-to-day logistics (Figure 1).

During the Melas, each patient encounter is typically recorded on a sheet of paper known as the “OPD” (outpatient department) paper, a near ubiquitous document in all state-run primary care clinics in India. The form used in Maharashtra has not changed since 1933 and is typically completed for the patient by the nurse and carried by the patient first to the doctor and then to the pharmacist, who retrieves the document while dispensing medication. Various parameters, such as medications dispensed and clinical diagnoses, are tallied manually and entered into a log by the nurses and pharmacists at the end of the workday. The log is then physically transported to a nodal office where similar reports from all the temporary clinics are totaled and entered into a spreadsheet that is then transmitted to other administrators. On busy days, the staff spend hours (over time) tallying the spreadsheets late in the night to provide actionable analysis the next morning.

The inefficiency and possible inaccuracy associated with the system prompted India’s National Disaster Management Authority to pilot a tablet-based system at the Allahabad Mela in 2013 [18].In 2015, the then principal secretary of public health in Maharashtra asked for the disease surveillance system to be digitized. Acknowledging the significant human labor associated with tallying the data manually every day, the local administrative team in Nashik was strongly in favor of digitizing the data, but skeptical about the participation of clinical staff. The clinicians were cautious and guarded about embracing a new digital tool, fearing that it would make their job harder.

### Step 2: Ideation

#### Common Themes

Affinity diagrams resulting from open-ended discussions with multiple stakeholders helped identify the following key themes: the tool would have to offer more than simply a digital medium to collect data; it would necessarily have to make the jobs of the clinical staff easier, not harder; it would have to save time; it would need to avoid redundancy; it would need to result in actionable, reliable, verifiable, and timely analysis; the clinicians entering the data would need to see the benefit; it would need to have little to no barriers for onboarding users; and adoption and retention would need to depend on the clinician’s user experience [36]. These observations led to the design decisions described below.

#### Data Minimization

The existing paper-based system relegated clinical and ancillary staff to duplicating a low-yield, inefficient, clerical task. In the absence of confirmatory tests, syndromic surveillance would only require daily incidence of presenting complaints. Given the dynamic population flux in and out of the city, a spike in absolute numbers could merely reflect a transient rise in the visiting population. For effective surveillance, the relative risk of one disease compared to the incidence of others would be helpful. The only data points that were strictly necessary for such syndromic surveillance were comprehensive tallies of all presenting complaints. Age, gender, and the location of the clinic would also be informative. Other information like patients’ social histories, the treatments they were given, and even their vital signs, while all essential for documenting a good clinical encounter, are unnecessary for syndromic surveillance, especially in a resource-constrained environment with little analytic or response capacity to use the additional information.

#### Prototyping

**Figure 2:**
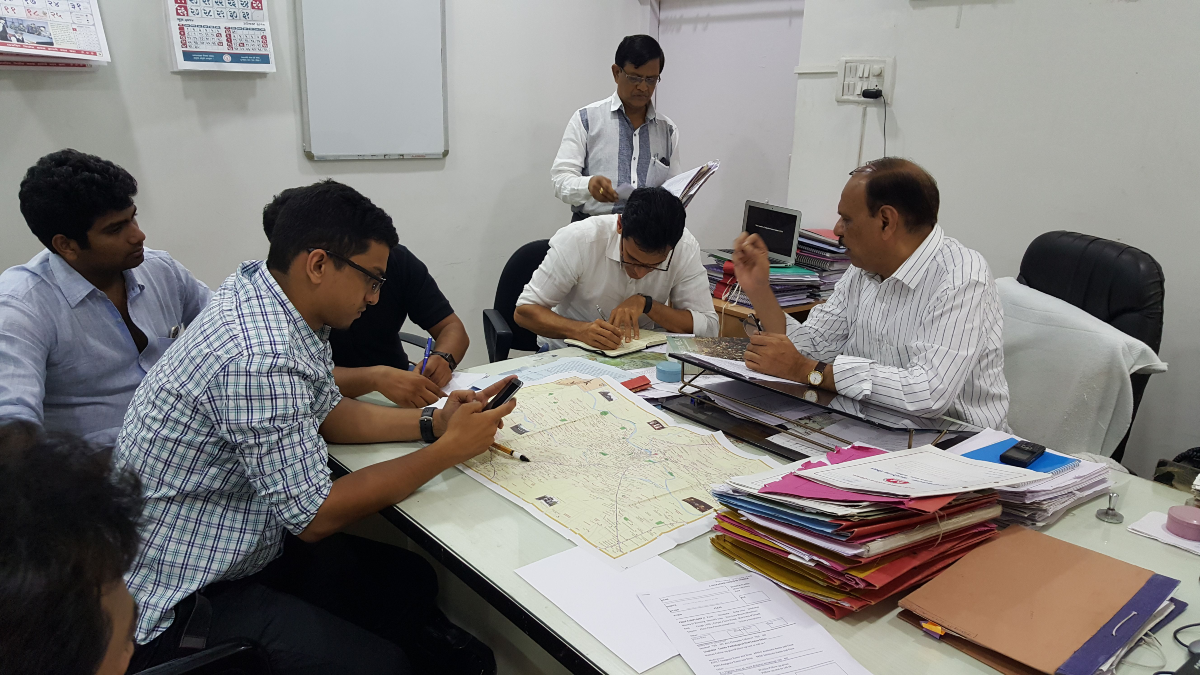
Understanding the layout, context, and disease surveillance needs from public health officials at the start of the Mela.

To find a solution that balanced fidelity, speed, and cost, this stage of the ideation process involved co-creation to ensure that end-user needs informed design choices (Figure 2) [16,37].

Historically, having only used tallied data from spreadsheets, local officials struggled to envision dashboard designs and requested a printable version of the “table” they were most familiar with. In order to overcome design fixation, we adopted parallel prototyping to develop the following 2 sets of outputs: a data-entry tablet-based interface and a visualization tool for public health officials.

#### Tablet Interface

**Figure 3:**
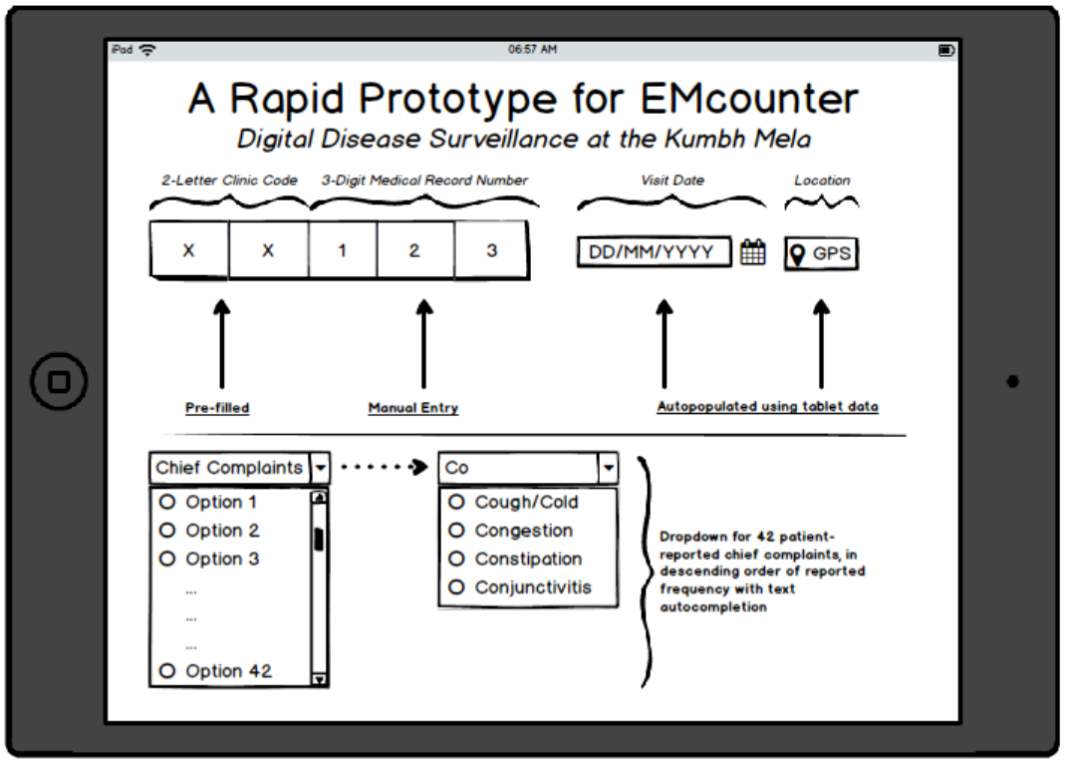
An initial mockup of the proposed EMcounter tool, minimizing data entry requirements for providers at the Mela while reducing errors in reporting for real-time epidemiological surveillance.

**Figure 4:**
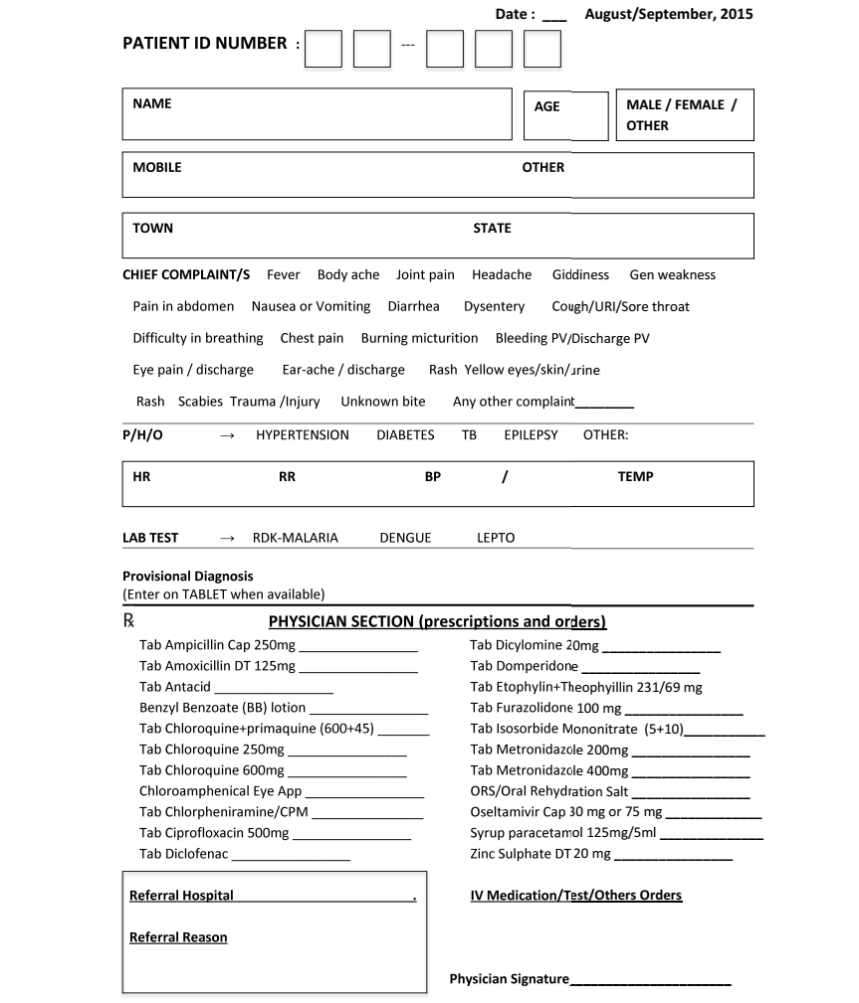
The OPD paper with structured sections and tailored response options, abbreviated from the free-text OPD sheet provided preintervention to providers at the Mela. OPD: outpatient department.

To optimize the tool’s user interface and user experience, clinicians who would staff the Mela clinics were involved in co-creating the data-entry tool. The design team met with over 100 doctors and nurses during their preparatory training sessions a month prior to the Mela. Following an orientation section that described the intervention at the 2013 Mela and the potential for real-time analytics for census, inventory, and disease surveillance, the clinical staff were offered usability testing in small groups. Users were invited to provide feedback on the perceived utility and usability of the tool, with particular attention to the interface (positioning; font type and size; and entry choices including checkboxes, radio buttons, steppers, toggle switches, dropdowns, and list boxes). This process informed iterative cycles of creation and testing. The inclusion of end users in this dynamic process helped to foster their sense of ownership in the process and ultimately resulted in high adoption rates. The final tool collected only 4 data points necessary for surveillance and clinical care (the medical record number [MRN], age, sex, and chief complaint). The MRN was structured such that it revealed clinic location and date, both of which were autopopulated in the digital tool, requiring the physician to only enter the last 3 digits, and this greatly minimized data-entry errors (Figure 3). A total of 42 presenting complaints were listed in a drop-down menu, in order of expected frequency, with autocompletion text options and with the buttons, font size, and positioning of modules tailored to physician needs. Date, time, and GPS data were autopopulated from the tablet data. This degree of attention to the user experience was necessary to reduce the number of clicks required of physicians and to reduce their cognitive burden [20]. The abbreviated OPD paper contained a field for the registration number, and a checklist of diagnoses and the most commonly prescribed medications (Figure 4).

#### Dashboards

In addition to the tables requested by the public health officials, the final product included downloadable tables (Multimedia Appendix 1), as well as a real-time interactive dashboard that allowed the user to query and filter the data via several permutations of location, customized timeframe, age group, gender, and chief complaint (Multimedia Appendix 1). This would allow real-time epidemiological exploration of any observed atypical trends, triggering the public health system to rapidly launch an inquiry or response.

#### Field Testing

**Figure 5:**
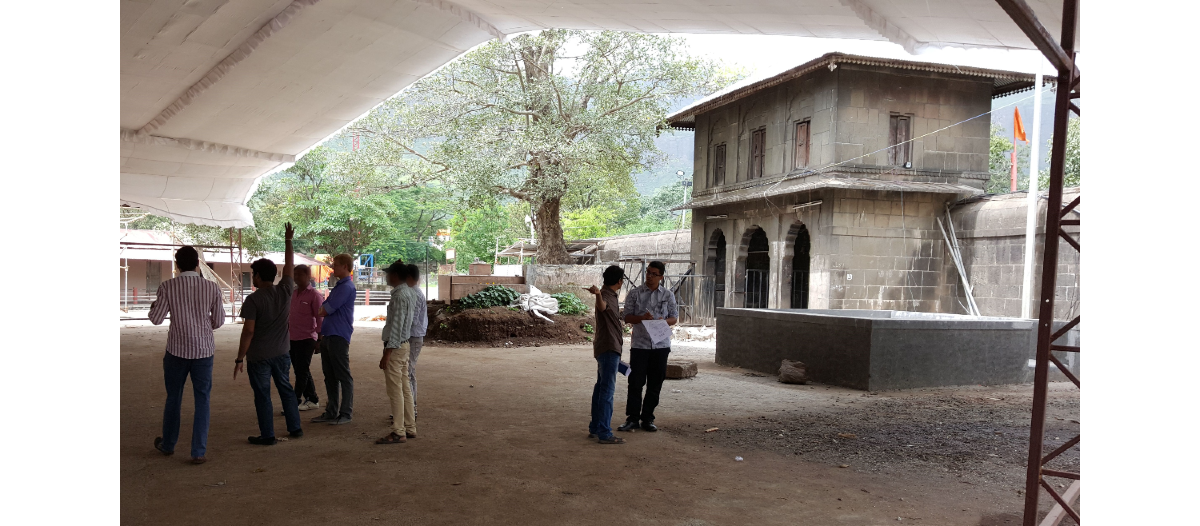
A pre-Mela site visit revealing that some clinics would be held in spaces with thick stone walls, precluding cellular service for real-time reporting of collected data.

The 50 temporary clinics across Nashik and Trimbakeshwar included temporary structures made from cloth, bamboo, and rope; repurposed rooms in existing buildings; and even designated areas in cavernous temple halls buttressed by thick stone walls, impermeable to cell phone signals (Figure 5). The system was designed to transmit data over 3G and 4G connections via SIM-enabled tablet computers to the cloud-based analytic tool, remotely accessible with authentication. The field visits revealed that several sites did not have good cell phone coverage, requiring that the devices be periodically swapped and brought to an area with good signal. The clinics had few power outlets, and they were often away from the doctor’s desk, necessitating that each tablet computer be accompanied by an extra battery pack and a multipoint extension cord.

### Step 3: Implementation

#### Preparation

**Figure 6:**
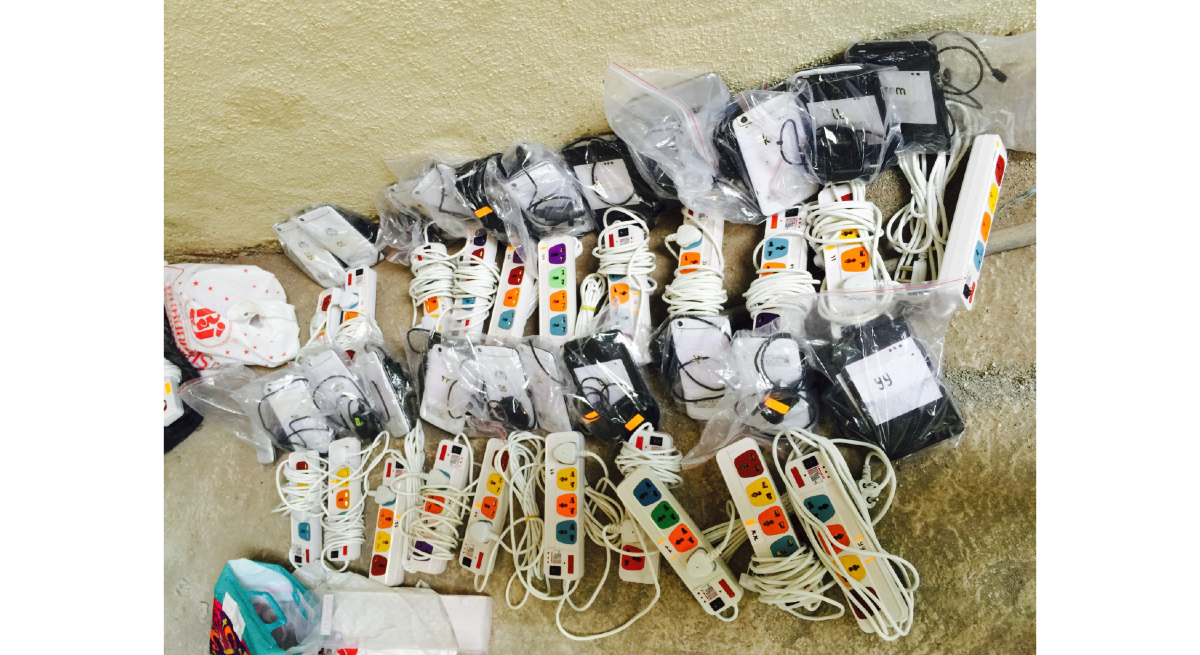
The EMCounter kit provided to each clinical team with labeled tablets, power supply, instructions for troubleshooting, and contact information of designated support team members.

The co-creation processes helped the design team preempt and address issues that normally account for poor user compliance, including poor perceived utility, lack of incentive, and suboptimal user experience. The ideation process and field visits allowed us to preempt a series of logistical and infrastructural issues, resulting in organized “deployment kits” for each clinic site. The kit included a battery pack, extension power cord, tablet computer, user manual, sign-out sheet, and phone number to a 24/7 helpline of medical student volunteers trained in troubleshooting the software (Figure 6).

#### Training and Support

**Figure 7:**
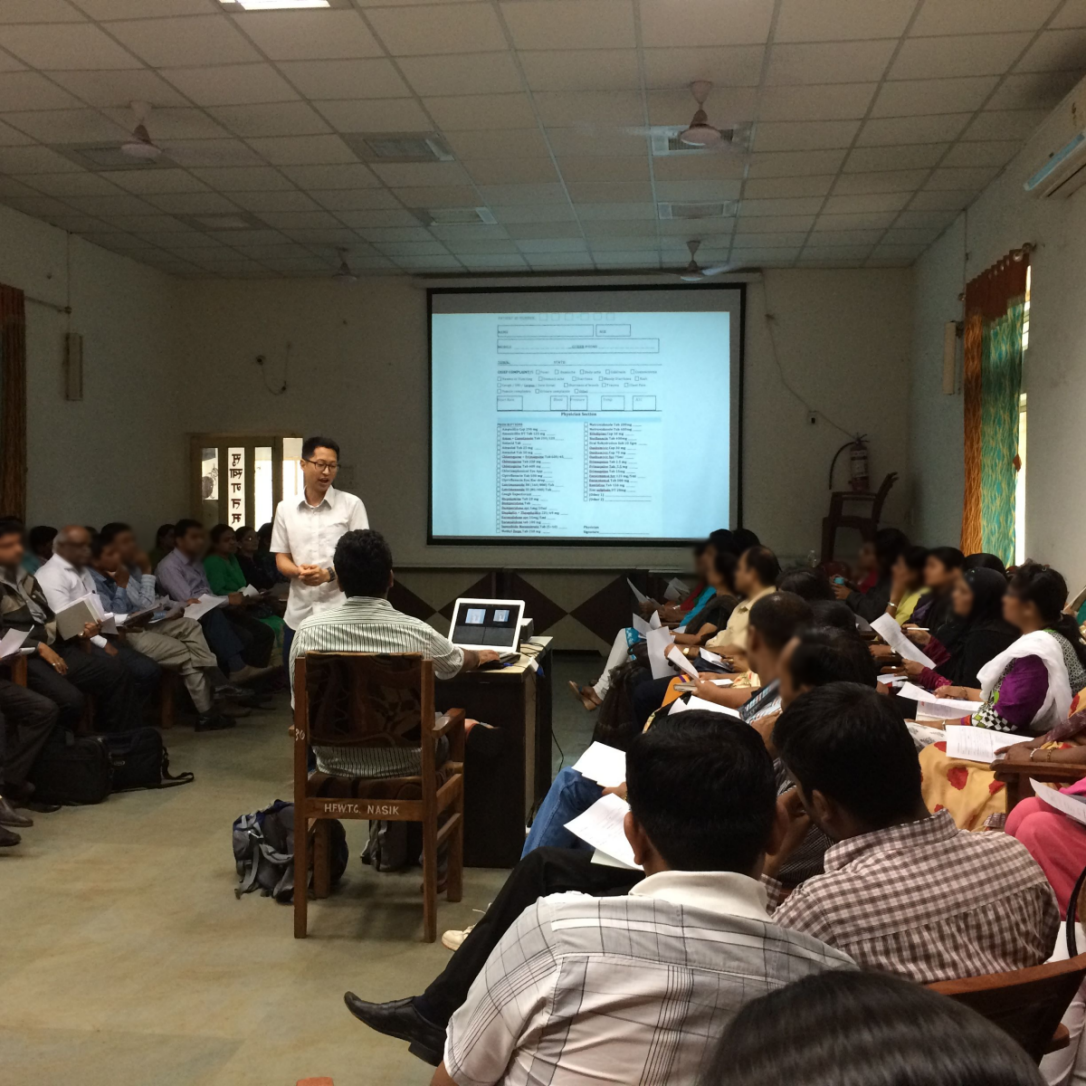
Core team member Dr John Won introduces the 2015 digital surveillance effort.

A 1-hour training session was allocated by the public health department for demonstrating and using the tool, on the eve of the Mela. Most of the physicians had seen earlier iterations during the co-creation sessions and were able to test the tool with little to no supervision (Figure 7). Many of the pharmacists and nurses did not receive any training, were unaware of the project, and were trained on-site the next morning.

**Figure 8:**
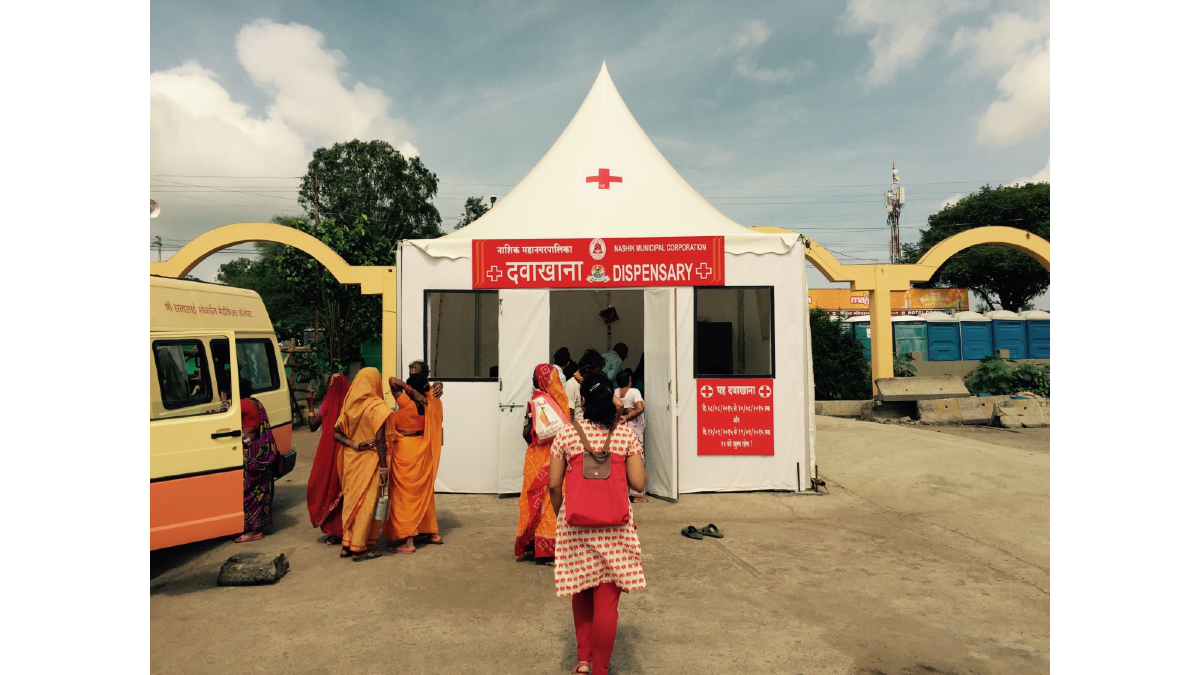
A volunteer medical student from the troubleshooting team visits a tent clinic at the Mela as part of daily in-person check-ins to maintain data quality and debug any issues with the tool.

**Figure 9:**
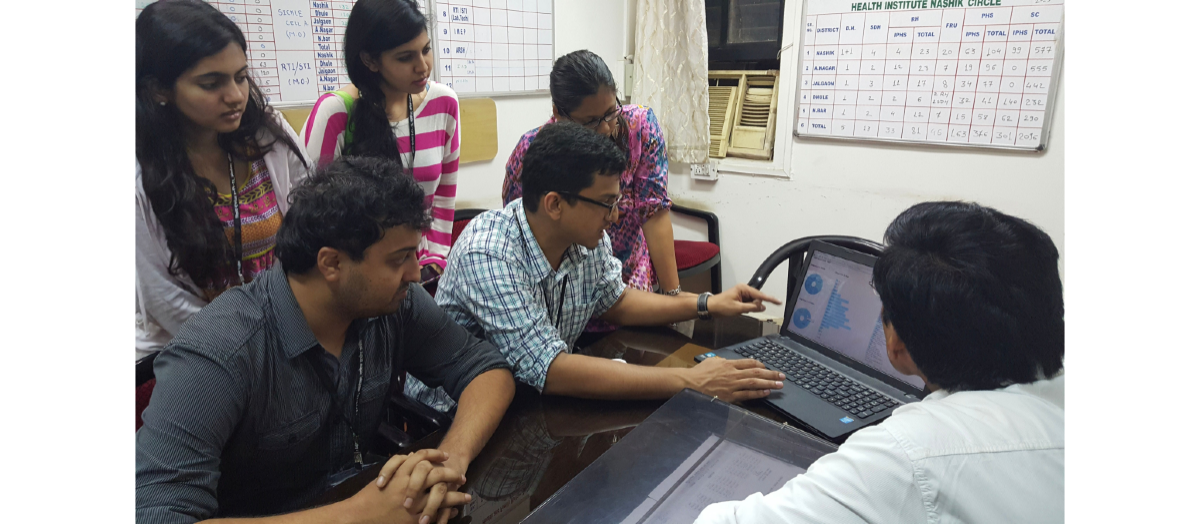
Core team members Dr Ahmed Shaikh and Dr Shashwat Hora interpret the real-time epidemiological data presented on dashboards, along with public health officials in Nashik.

The troubleshooting team comprised voluntary medical students (from India) and medical residents (from New York), all of whom had co-created the tool, and several of whom had worked at the 2013 Kumbh Mela and could anticipate the uncertainties associated with deployment in a large chaotic mass gathering. The government endorsed the software as the “official” data collection tool and circulated an official state memo to all providers mandating that they adopt the tool. Pairs of volunteers visited every site, twice daily, and checked in with every provider via phone (Figure 8). WhatsApp groups were used to send out checklists for every shift, and solicit daily and exit feedback. A core team stationed at a nearby hotel with a good Wi-Fi–based internet connection was in constant communication with the technical team in Boston and Mumbai, and with public health officials in Nashik and Mumbai, providing frequent updates and helping public health officials interpret and navigate the dashboard if necessary (Figure 9). During the site visit, clinicians were provided time to explore the dashboard to examine disease epidemiology, clinic population demography, and their own census compared to their peers.

#### Deployment

The EMcounter digital surveillance system was deployed at over 40 clinics across Nashik and Trimbakeshwar, and recorded 33,305 discrete patient encounters over 9 days. Compliance reached 100% by the end of the first day. Local public health officials learned to query the online dashboard and routinely consulted the core team for clarifications. On day 3, noticing a spike in diarrheal diseases at a particular clinic, public health officials dispatched a team of sanitation engineers to test water at all surrounding taps for contamination. A candid testimonial about the incident from one of the clinicians is available in Multimedia Appendix 2.

Mid-project feedback revealed that 100% of the users found value in the exercise and the analytics, and the ancillary staff stopped tallying paper records after initially comparing manually maintained logs to the reported data. The census data were particularly useful in addressing supply-demand mismatches, as not all clinics saw equal footfall despite being initially staffed uniformly

## Discussion

Digital disease surveillance has now been deployed by our team at 2 of the world’s largest mass gatherings, under conditions of extreme uncertainty and chaos. We attribute the tool’s success in 2015 to strict adherence to design principle standards. The interdisciplinary nature of our team that included public health practitioners, physicians, computer scientists, an embedded journalist, a filmmaker, a senior bureaucrat, and several end users, precluded early design fixation and allowed us to draw upon our expertise and the carefully implemented ideation process to generate a variety of prototypes for the final users to choose from. Co-creation also instilled a sense of buy-in from an otherwise underinvested group of clinicians who were redeployed for this job from across the state. Most importantly, taking the time to explain the epidemiological rationale for the intervention encouraged buy-in. This is seldom done with digital health programs that are rolled out at scale in either the public or private sector. The lack of co-creation and the absence of user buy-in and ownership result in poor compliance and rapid attrition, incorrectly leading to the conclusion that health care providers are resistant to change [37,38]. Data minimization will also become increasingly important as recent advances in India’s digital health ecosystem are likely to spur the collection of vast amounts of data. Data minimization, in addition to improving the provider experience, is also a sound privacy-preserving strategy [39,40].

This project had 2 significant limitations that may have further precluded large-scale adoption of the tool. There was no mandate to adopt any interoperability standards, as the project, despite being government sanctioned, was perceived by some as an external academic intervention. Since there were no existing integrated medical records in the public sector, this limitation was less consequential. Despite its significant success in meeting its articulated objectives, this project suffered from “pilotitis,” a common fate of digital health interventions everywhere [37]. The intervention was not expanded to all primary care sites in the state as was originally envisioned. A lack of institutional memory is particularly heightened in India due to the rapid turnover of officials. Table 1 compares the project’s performance against standards and principles for successful digital health implementation recommended by the recently published World Health Organization’s Digital Implementation Investment Guide [21].

**Table 1:** Comparison of EMcounter at the Kumbh Mela, implemented in 2015, with the World Health Organization’s Digital Implementation Investment Guide Checklist, released in 2020.

World Health Organization’s Digital Implementation Investment Guide	Correlation	Comment
Design with the user	High	Co-created with end users
Understand the existing ecosystem	High	Embedded team members
Design for scale	High	Light back-end and low-footprint technology used
Build for sustainability	Low	Poor buy-in from some stakeholders; lack of interoperability standards would hamper integration
Be data driven	High	Statistically sound analytics
Use open standards, open data, open source, and open innovation	Medium	Interoperability standards not adopted
Reuse and improve	High	Built on innovations in a prior Mela
Address privacy and security	High	Data anonymized at source
Be collaborative	High	Multidisciplinary international team

### Conclusion

Digital interventions fail when they ignore the complexity of health care interventions [36]. Unlike other sectors, there is wide variation in clinical practice from provider to provider, an even greater variation in workflow and routine among health care sites, and vast differences in health-seeking behaviors among patients, which are influenced by socioeconomic conditions, gender, age, and health literacy [41-43]. It is therefore imperative that investments in digital health projects underscore the need to co-create and pilot interventions before sanctioning their use at scale. Sandboxing, monitoring, and impact evaluation should be integral components of early design [44].

The plethora of digital applications, especially digital contact tracing solutions, adopted and deployed during the pandemic by governments around the world, despite little evidence that they work, highlights the importance of a deliberate thoughtful approach to deploying such interventions. There is little reason to not hold digital tools to the same high standard of scientific rigor as any other public health intervention.

## Appendix

Multimedia Appendix 1 The EMcounter dashboard.

Multimedia Appendix 2 Clinician testimonial about the use of EMcounter for syndromic surveillance at the Kumbh Mela.

## Abbreviations

MRN: medical record number
NDHM: National Digital Health Mission
OPD: outpatient department
